# Occurrence of plastidial triacylglycerol synthesis and the potential regulatory role of AGPAT in the model diatom *Phaeodactylum tricornutum*

**DOI:** 10.1186/s13068-017-0786-0

**Published:** 2017-04-20

**Authors:** Srinivasan Balamurugan, Xiang Wang, Hong-Lei Wang, Chun-Jing An, Hui Li, Da-Wei Li, Wei-Dong Yang, Jie-Sheng Liu, Hong-Ye Li

**Affiliations:** Key Laboratory of Eutrophication and Red Tide Prevention of Guangdong Higher Education Institute, College of Life Science and Technology, Jinan University, Guangzhou, 510632 China

**Keywords:** AGPAT, Triacylglycerol, Diatom, Biofuels

## Abstract

**Background:**

Microalgae have emerged as a potential feedstock for biofuels and bioactive components. However, lack of microalgal strains with promising triacylglycerol (TAG) content and desirable fatty acid composition have hindered its commercial feasibility. Attempts on lipid overproduction by metabolic engineering remain largely challenging in microalgae.

**Results:**

In this study, a microalgal 1-acyl-sn-glycerol-3-phosphate acyltransferase designated AGPAT1 was identified in the model diatom *Phaeodactylum tricornutum*. AGPAT1 contained four conserved acyltransferase motifs I–IV. Subcellular localization prediction and thereafter immuno-electron microscopy revealed the localization of AGPAT1 to plastid membranes. AGPAT1 overexpression significantly altered the primary metabolism, with increased total lipid content but decreased content of total carbohydrates and soluble proteins. Intriguingly, AGPAT1 overexpression coordinated the expression of other key genes such as DGAT2 and GPAT involved in TAG synthesis, and consequently increased TAG content by 1.81-fold with a significant increase in polyunsaturated fatty acids, particularly EPA and DHA. Moreover, besides increased lipid droplets in the cytosol, ultrastructural observation showed a number of TAG-rich plastoglobuli formed in plastids.

**Conclusion:**

The results suggested that AGPAT1 overexpression could elevate TAG biosynthesis and, moreover, revealed the occurrence of plastidial TAG synthesis in the diatom. Overall, our data provide a new insight into microalgal lipid metabolism and candidate target for metabolic engineering.

**Electronic supplementary material:**

The online version of this article (doi:10.1186/s13068-017-0786-0) contains supplementary material, which is available to authorized users.

## Background

Microalgal triacylglycerols (TAGs) have garnered significant attention as sustainable feedstock for biofuels and bioproducts. Functional and economic characteristics of TAGs are determined by fatty acid constituents and their stereospecific distribution on glycerol backbone, such as polyunsaturated fatty acids (PUFAs), the components of much interest due to their well-established health benefits in humans [1]. However, the diverse nature of microalgal lipid composition hindered their commercial potential. As such, there is considerable interest in microalgal metabolic engineering for TAG overproduction with desired fatty acid profile. Nevertheless, the molecular mechanisms that underlie the TAG synthesis remain elusive in oleaginous diatoms.

In microalgae, TAG is considered to be synthesized by the endoplasmic reticulum (ER)-localized pathway via sequential esterification of glycerol backbone at the sn-1, sn-2, and sn-3 by glycerol-3-phosphate acyltransferase (GPAT), lysophosphatidate acyltransferase (LPAT/AGPAT), and diacylglycerol acyltransferase (DGAT), respectively [2–4]. However, an alternate plastidial TAG biosynthetic mechanism was proposed in *Chlamydomonas reinhardtii* and demonstrated the storage of resultant TAG in lipid droplets in both cytosol and plastid [4]. The acyl chain composition of fatty acids is facilitated by the narrow and broad substrate specificity of acyltransferases of de novo TAG biosynthetic pathway [5–7]. Among the three acyltransferases involved in TAG synthesis, GPAT and DGAT have broader acyl-CoA substrate specificity in contrast with the stringent substrate specificity of AGPAT towards particular acyl-CoAs [8]. The abundance of unusual C18 fatty acids with unsaturation at the sn-2 position in most of plant seed oils was due to the narrow acyl-CoA substrate specificity of AGPAT, which catalyzes the second acylation at the sn-2 position of glycerol backbone [7]. As the positional distribution of fatty acids is based on the diverse substrate specificity of these enzymes, AGPAT with pronounced substrate specificity have garnered significant attention for engineering microalgae with desired fatty acid composition.

Based on the subcellular localization, AGPATs are classified into plastid- and ER-localized AGPAT. The former has a substrate preference for 16:0-ACP over 18:1 ACP and the latter show substrate preference for 18:1 over 16:0 CoA [6, 9]. The characterization of the pronounced substrate specificity of AGPAT isoforms has been documented in mammals [10, 11], microalgae such as *C. reinhardtii* [12], and in several plant species including *Arabidopsis thaliana *[5, 6], *Echium pitardii* [7], *Crambe abyssinica* [8], and *Cocos nucifera* [13, 14]. However, the identification and functional characterization of AGPAT in diatoms remain largely unknown. Here, we identified an AGPAT in oleaginous diatom *P. tricornutum* and revealed its role in lipogenesis and the existence of plastidial TAG biosynthetic pathway in addition to ER-located pathway.

## Methods

### Microalgal strain and culture conditions

*Phaeodactylum tricornutum* (No: CCMP-2561) was purchased from the Provasoli-Guillard National Center for Marine Algae and Microbiota, USA. *P. tricornutum* was cultivated in f/2 medium without Na_2_SiO_3_·9H_2_O and grown at 21 ± 0.5 °C in an artificial climate incubator, under a 12:12 h light/dark photoperiod provided by cool white fluorescence light with 200 μmol photons m^−2^·s^−1^ irradiance. Microalgae density was determined by direct count with a Neubauer Brightline hemocytometer under light microscopy every day.

### Gene cloning, construction, and transformation

Amino-acid sequence of *P. tricornutum* AGPAT1 were retrieved from the National Center for Biotechnology Information (NCBI, https://www.ncbi.nlm.nih.gov/). The amino-acid sequences of AGPAT/LPAT proteins from four species were aligned by ClustalX2. Phylogenetic tree was constructed using MEGA7 by neighbor-joining algorithm based on the amino-acid sequences of GPAT and AGPAT from *Brassica napus*, *Arabidopsis thaliana,* and *P. tricornutum*. Subcellular localization of AGPAT1 was predicted using the online tools such as WoLF PSORT and LocTree3.

Total RNA of *P. tricornutum* was extracted by Plant RNA kit (Omega, USA) and transcribed into DNA using HiScript 1st Strand cDNA Synthesis Kit (Vazyme, China) following the manufacturer’s instruction. The coding region of *AGPAT1* was PCR amplified using the primers AGPAT1-F and AGPAT1-R (Table 1).

**Table 1:** Primers and their sequences used in this study

Primer	Sequence (5′ → 3′)
AGPAT1-F	ACAATTACAATCCAGTGGTACCATGAGGCATTTGAGAGGCGTAC
AGPAT1-R	GAGTTTTTGTTCCAGGTGTGGTACAGTAGTCTCCTCCGT
CAT-F	ATGGAGAAAAAAATCACTGGATATACC
CAT-R	TTACGCCCCGCCCTGCCACT
AGPAT1-qF	TACCGATATGATGGAGATGG
AGPAT1-qR	AGACTACCTTATTACCTTGGG
GPAT-qF	ACGACAAGGTCGGAACAAAC
GPAT-qR	TAAAGGCACCGTCCTTGAAC
DGAT2-qF	GATCTGGCCTAAATCCGTCA
DGAT2-qR	CGACGATGAGACGATCAAGA
ACT1-F	AGGCAAAGCGTGGTGTTCTTA
ACT1-R	TCTGGGGAGCCTCAGTCAATA

**Fig. 1:**
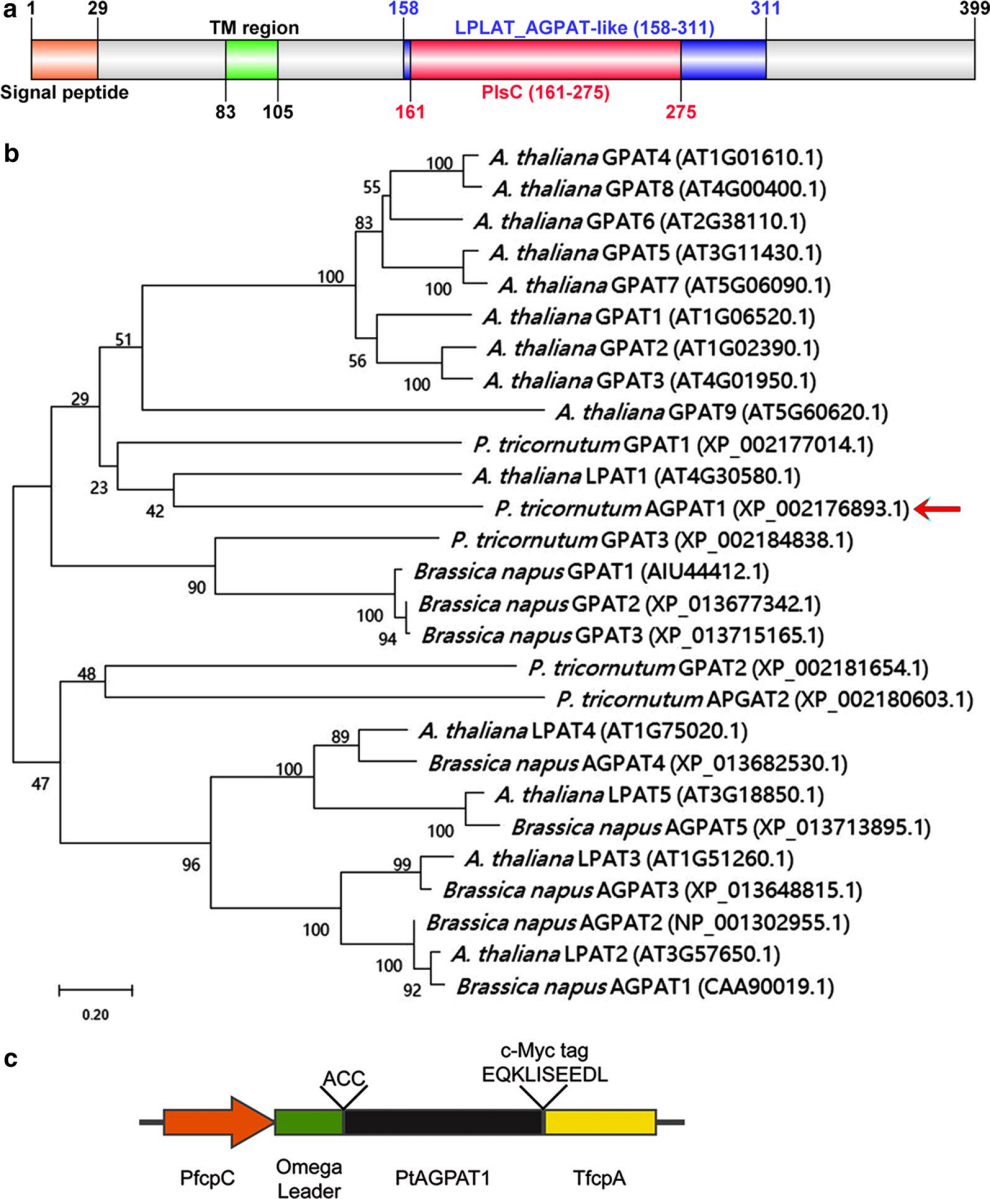
Conserved domains, phylogenetic analysis, and construct map of AGPAT1. **a** Schematic distribution of conserved domains in AGPAT1 analyzed by NCBI. **b** Phylogenetic relationship of AGPAT1 with the known AGPATs from various organisms. The phylogenetic tree was constructed from a complete alignment of 27 proteins MEGA7 using neighbor-joining method. The optimal tree with the sum of branch length = 13.14606879 is presented. The percentages of replicate trees in which the associated taxa clustered together in the bootstrap test (500 replicates) are represented along the branches. All positions containing gaps and missing data were eliminated. The arrow indicates AGPAT1 of *P. tricornutum.* **c** Schematic map of AGPAT1 expression cassette

The fragment of *AGPAT1* was purified by TaKaRa MiniBEST DNA Fragment Purification Kit (Takara, Japan) and the *PtAGPAT1* coding sequence was cloned in between the fucoxanthin chlorophyll a/c binding protein (fcp) *PfcpC* promoter and *TfcpA* terminator in the expression vector pHY-18 [15] by ClonExpress II one step kit (Vazyme, China) according to the manufacturer’s protocol. An Omega leader nucleotide motif was included upstream of *PtAGPAT1* to enhance the translation. The schematic drawing of recombinant expression cassette pHY18-AGPAT1 was shown in Fig. 1c. The recombinant plasmid was electroporated into *P. tricornutum* using a GenePulser Xcell apparatus (Bio-Rad, USA) as described previously [16].

The transformants were cultured in f/2 liquid medium in darkness for at least 48 h. Thereafter, the cells were harvested and spread onto the plate containing f/2 solid medium supplemented with chloramphenicol (250 mg/L). After 3–4 weeks, surviving colonies were picked up and inoculated into fresh f/2 liquid medium containing chloramphenicol (250 mg/L). Transformed microalgae were sub-cultured once a week. Microalgae during the stationary phase cultivated without chloramphenicol were employed for the further analyses.

### Molecular analysis of transgenic microalgae

To detect the integration of the expression cassette in transformed microalgae, genomic PCR was performed. DNA of transgenics and wild type (WT) was isolated using HP Plant DNA kit (Omega, USA). PCR was performed to amplify the integrated *CAT* gene existed in the recombinant expression vector using genomic DNA as the template with the CAT primers (Table 1).

To detect the relative transcript abundance of *AGPAT1* in microalgae, quantitative real-time PCR (qPCR) was determined with an AceQ qPCR SYBR Green Master Mix (Vazyme, China) and performed on CFX96 real-time PCR detection system. Total RNA from transgenics and WT was extracted with Plant RNA Kit (Omega, USA) and reversely transcribed into cDNA with HiScript II Q RT SuperMix for qPCR (Vazyme, China) according to the manufacturer’s instructions. qPCR was performed following the standard method as previously described [17]. The relative transcript level of *AGPAT1* was determined by the 2^−ΔΔCt^ method after normalization to the endogenous control gene *β*-*actin*. Three biological replicates were performed. All qPCR primers were listed in Table 1.

To detect the expression of recombinant protein in transgenics, Western blot analysis was carried out against c-Myc-tag. Microalgae during stationary phase were harvested and the total protein was extracted using RIPA Lysis Buffer (Beyotime, China) added with PMSF. Protein concentration was measured using BCA Protein Quantification Kit. First, proteins were separated by SDS-PAGE (sodium dodecyl sulfate–polyacrylamide gel electrophoresis) and electrotransferred onto a polyvinylidene difluoride (PVDF) membrane. The membrane was blocked by skim milk for 1 h at 4 °C and then incubated for overnight with primary anti-c-Myc antibody (1:3000, Abcam, UK) at 4 °C. The membrane was then washed thrice with phosphate buffer solution with Tween-20 (PBST) and thereafter incubated with HRP-conjugated goat anti-rabbit secondary antibody (1:5000, CST, USA) for 2 h at 4 °C. The membrane was developed by chemiluminescence system. Endogenous β-actin protein was used as internal reference.

Enzymatic activity of AGPAT in transgenics and WT was measured using the total AGPAT activity spectrophotometry assay kit following the manufacturer’s instruction (Genmed, China).

To evaluate the impact of AGPAT1 overexpression on TAG synthesis, the relative transcript abundance of several crucial genes involved in TAG synthesis was also determined by qPCR. The qPCR was carried out by following the same protocol as described above. A complete list of all primers used in this study and their sequences were given in Table 1.

### Photosynthetic efficiency

The chlorophyll fluorescence parameter Fv/Fm (ratio of variable/maximum fluorescence) of microalgae was analyzed with phytoplankton analyzer (WALZ, Germany) following the protocol as described previously [18].

### Lipid analysis

The neutral lipid content of microalgae was determined by Nile red (Sigma, USA) staining as described [19]. An aliquot of 3 mL algal culture was added with 30 μL of Nile red (0.1 mg/mL, dissolved in acetone) and lucifugally incubated at 37 °C. The unstained microalgae, stained microalgae, and stained natural seawater were added into 96-well plate. The relative fluorescence intensity of neutral lipid was detected at an excitation wavelength of 530 nm and an emission wavelength of 592 nm on the microplate reader (Bio-Tek, USA).

Total lipids from microalgae were extracted according to the modified method of Bligh and Dyer [20] and the lipid content of cell dry weight was determined gravimetrically. Briefly, freeze-dried microalgae were ground to a fine powder and extracted with 3.8 mL of methanol/chloroform/water (2:1:0.8, *v/v*) with sonication. After sonication, the content was augmented with 2 mL of water/chloroform (1:1, v/v). The content was vortexed and centrifuged at 1500 g for 5 min to separate the mixture. The upper phase (aqueous phase) was discarded and the lower phase (chloroform) was collected and concentrated under N_2_ stream.

Total lipids were fractionated into neutral lipids (NLs), phospholipids (PLs), and glycolipids (GLs) using solid-phase extraction (SPE) through gravity with pre-packed silica cartridges (500 mg, 6 cc Sep-Pak, Waters, USA) as described by Li et al. [21]. The fractionated lipids were then determined gravimetrically.

Fatty acid composition was analyzed as fatty acid methyl esters (FAMEs) using gas chromatography–mass spectrometry (GC–MS). Briefly, 500 μL toluene was added to the wet algae pellet (about 5 mg) and transferred to a Teflon-lined screw-cap tube, then 1 mL fresh 0.5 N NaOH/MeOH was added. The mixture was vortexed and incubated at 80 °C for 20 min. After cooling at room temperature for 5 min, 1 mL fresh AcCl/MeOH (1:10, v/v) was slowly added and incubated for further 20 min. Then, 1 mL 6% K_2_CO_3_, 500 μL hexane, and 10 μL methyl nonadecylate (Aladdin, China) were added and vortex for 1 min. The upper phase was collected for GC–MS. TAG was separated by thin-layer chromatography (TLC) using a hexane/diethyl ether/acetic acid solvent system (85:15:1, v/v) on a silica plate using a glyceryl triheptadecanoate standard (Sigma, USA). The separated TAGs were visualized under iodine vapor and subsequently transmethylated into FAMEs, and the fatty acid composition was analyzed by GC–MS.

### Measurement of cellular primary metabolites

To explore the changes in primary metabolism by AGPAT overexpression, total carbohydrate and total soluble protein content were determined. The carbohydrate content was determined following the phenol–sulfuric acid method [22]. Briefly, microalgae (~50 mL) were harvested and resuspended in 1 mL water. The above suspension was further added with 1 mL 5% phenol solution (w/v). Then, 5 mL of sulfuric acid (95–98%, v/v) was rapidly added into the mixture solution without any drops on the wall and then incubated at 25 °C for 10 min. Thereafter, the mixture was incubated in water bath for 20 min at 30 °C. The solution would turn to orange color as the phenol reacted with carbohydrates and the color could be detectable at 483 nm. Glucose was used as the standard and the concentration was up to 1 g L^−1^. The protein content was determined as mentioned above in Western blot assay.

### Confocal microscopy and subcellular localization of AGPAT1

Microalgal cells were stained with Nile red and incubated in darkness for 20 min at 37 °C. Stained cells were observed under a laser-scanning confocal microscope Zeiss LSM510meta (Zeiss, Germany) with an excitation wavelength of 488 nm and an emission wavelength of 505–550 nm. Subcellular localization of AGPAT1 was further assessed by immuno-electron microscopy (EM) as previously described (JEM-2000EXII JEOL, Japan) operating at 80 kV [2]. All samples for EM analysis were prepared at South China Botanical Garden, Chinese Academy of Sciences.

### Statistical analysis

All the experiments were performed in triplicate and the mean ± standard deviation (SD) values were calculated for each. The statistical significance of the difference was tested using Student’s *t* test and indicated as *p* < 0.05 (*) or *p* < 0.01 (**).

## Results and discussion

### Sequence analysis of AGPAT1 of *P. tricornutum*

The gene annotated as 1-acyl-sn-glycerol-3-phosphate acyltransferase in the genome sequence of *P. tricornutum* was designated AGPAT1 in this study (PHATRDRAFT_43099, GenBank accession: XP_002176893.1). Conserved domains of AGPAT1 were identified by NCBI and shown to belong to the LPLAT superfamily which exists in various organisms (Fig. 1a). A plsC domain was also identified in AGPAT1 by SMART which also exists in bacterial AGPAT (Fig. 1a). Phylogenetic analysis of amino-acid sequences of GPAT and AGPAT from several organisms showed that *P. tricornutum* AGPAT1 shared the highest similarity with AtLPAT1, RcLPAT1, and CpuLPAT1 (Fig. 1b; Additional file 1: Figure S1a). Interestingly, in *P. tricornutum*, GPAT1 also showed some consanguinity with AGPAT1. In AGPAT1, motifs of the acyltransferase consist of the conserved sequences VANHASWLDI (residues 164-173, motif I), NHILIDR (residues 206-212, motif II), FPEGMRSRDGKL (residues 238–249, motif III), and VPIVPITI (residues 265–272, motif IV) (Additional file 2: Table S1), with motifs III and IV the most conserved (Additional file 1: Figure S1b). Various motifs were predicted to exist in the acyltransferase domain, among them were found to be conserved in diverse organisms, and the motifs I-IV were reported to be pivotal for the enzymatic activity of acyltransferase [5, 11, 23, 24]. In human, all the four conserved motifs were found to play a crucial role in AGPAT catalysis [11]. Comparison of PtAGPAT1 motifs with other species showed closer conservation in motifs III and IV, whereas the motifs I and II were conserved but did not show close homology with the reported plant AGPATs (Additional file 1: Figure S1b). Mutagenic analysis of all the four conserved motifs of human AGPAT revealed the invariant amino-acid residues in the motifs are necessary for acyltransferase activity [11]. The motif II with positive charge amino acid was characterized as hydrophobic LPA binding site. Site-directed mutations led to replacement of arginine resulted in loss of enzymatic activity, revealed the importance of positive charged amino acid in AGPAT activity [11]. In LPAT of *Cuphea avigera var. pulcherrima* and *C. viscosissima*, the existence of hydrophobic amino-acid residues was reported to determine the medium acyl chain length substrate for LPAT [5]. These reports suggest the role of distinctive hydrophobic amino-acid-rich region in motif II of AGPAT1 in recognizing the hydrophobic fatty acids with medium acyl chain. Further mutational studies in each conserved motif of AGPAT1 may help to elucidate the substrate specificity characteristics. Existence of transmembrane domain in AGPAT1 was predicted using three transmembrane prediction programs such as SOSUI version 1.11 (http://harrier.nagahama-i-bio.ac.jp/sosui/sosui_submit.html), TMHMM version 2.0 (http://www.cbs.dtu.dk/services/TMHMM/), and HMMTOP version 2.0 (http://www.enzim.hu/hmmtop/html/submit.html). Surprisingly, SOSUI prediction analysis revealed the presence of two transmembrane domains, whereas TMHMM and HMMTOP showed only one transmembrane domain (Additional file 1: Figure S2a). Two topologies of AGPAT1 were predicted according to the results obtained from Additional file 1: Figure S2a. As shown in Additional file 1: Figure S2b, SOSUI revealed that the N-terminal of AGPAT1 was predicted to be presented inner part of chloroplast and motif I existed within TM2. Other three motifs (II, III, and IV) existed inside the chloroplast. As shown in Additional file 1: Figure S2c, TMHMM and HMMTOP revealed the existence of single transmembrane and all the four motifs existed in the cytosol.

### Characterization of transgenic microalgae by molecular approaches

**Fig. 2:**
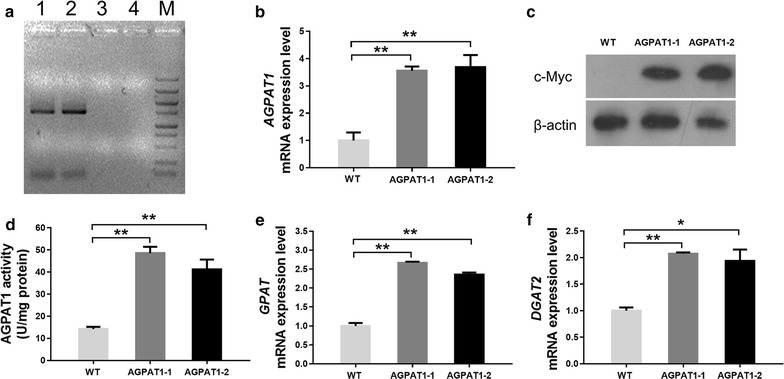
Molecular characterization of AGPAT1-overexpression microalgal transformants. **a**, PCR amplification of *CAT* gene from the genomic DNA of transformants. *Lanes 1* and *2*: transgenic lines, *Lanes 3* and *4*: negative control (wild-type genomic DNA and water, respectively); *Lane M* 5000 bp ladder marker. **b** Relative transcript abundance of *AGPAT1* determined by qPCR. The results were normalized against *β-actin* gene. **c** AGPAT1 protein expression measured by Western blot analysis with anti-c-Myc antibody. β-actin was used as an internal control. **d** Enzymatic assay of AGPAT1. **e** Relative transcript level of GPAT by qPCR. The results were normalized against *β-actin* gene. **f** Relative transcript level of DGAT2 by qPCR. The results were normalized against β-actin gene. Significant difference between wild type and transgenic microalgae is indicated at *p < 0.05 or **p < 0.01 level. Each value represents mean ± SD (*n* = 3)

Genomic PCR analysis was performed to detect the integration of chloramphenicol acetyltransferase (CAT) sequence. As expected, 0.7-kb amplicon was amplified by genomic PCR in transgenic lines, whereas no such band was detected in wild type (WT) (Fig. 2a). This result revealed that the expression cassette had been integrated into the genome of *P. tricornutum*. The relative transcript abundance of AGPAT1 in transgenics was determined by qPCR which showed that transcript level of PtAGPAT1 was dramatically increased by 3.55- and 3.69-fold in transgenic lines-1 and -2 compared to WT, respectively (Fig. 2b). A c-Myc tag was fused to the C-terminal of AGPAT1 in the expression cassette for detection of protein expression. Western blot analysis using anti-c-Myc antibody showed that a specific cross reactive protein band with expected molecular weight (46-kDa) was observed in transgenics, while no such band in WT (Fig. 2c). To further determine the activity of overexpressed AGPAT1 in transgenic lines, in vitro AGPAT1 assays were carried out from transgenic and WT lines. AGPAT1 activity was remarkably increased in transgenic lines than that of WT (Fig. 2d). These results showed that the expression cassette harboring AGPAT1 was successfully integrated, transcribed, and expressed in transgenic lines.

### AGPAT1 overexpression did not impair growth rate and photosynthetic activity

**Fig. 3:**
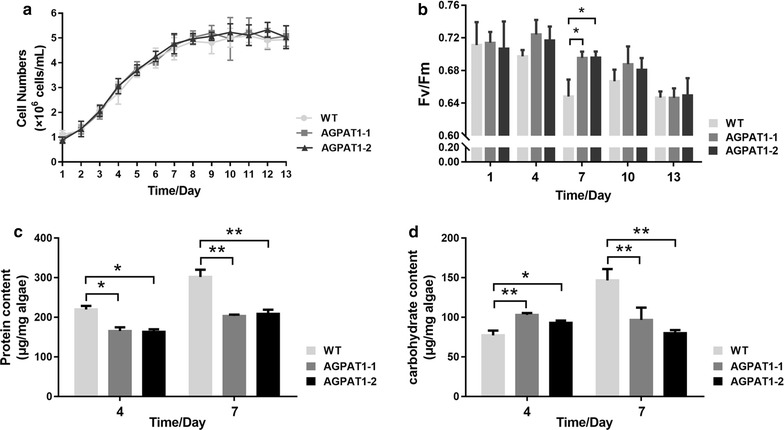
Analysis of cell growth, photosynthetic efficiency, and primary metabolites of *P. tricornutum*. **a** Growth curves. **b** Photosynthetic efficiency during the whole growth cycle. **c**, **d** Protein (**c**) and carbohydrate (**d**) content during middle exponential growth phase (day 4) and early stationary phase (day 7). Significant difference between WT and transgenic microalgae is indicated at *p < 0.05 or **p < 0.01 level. Each value represents mean ± SD (*n* = 3)

To analyze the impact of AGPAT1 overexpression on cellular and physiological conditions of transgenic lines, cell growth and photosynthetic efficiency were determined. Growth curve analysis showed that both the transgenics and WT exhibited similar growth pattern (Fig. 3a) and reached the stationary phase at the same period. This implied that AGPAT1 overexpression did not show adverse effect on microalgal growth. The chlorophyll fluorescence parameter Fv/Fm, the maximum quantum yield of photosystem II, has been considered as the sensitive parameter to evaluate photosynthetic performance. The value of Fv/Fm was dramatically increased in the transgenics than that of WT (Fig. 3b). Cells with higher Fv/Fm value imply the stable physiological status of the cell without being impaired by photoinhibition [25]. The previous studies have reported that provision of fatty acid precursors and protection of photosynthetic apparatus could enhance photosynthetic performance [2, 26, 27]. It is well known that AGPAT1 plays a significant role in producing phosphatidic acid (PA), a key fatty acid component in the synthesis of plastidial membrane, lipids etc. [3, 28]. Thus, the key role of providing fatty acid components by AGPAT1 resulted in elevated photosynthetic activity, which is in accordance with the previous reports.

### AGPAT1 overexpression altered the content of primary metabolites

To correlate the AGPAT1 overexpression with the synthesis of cellular components, total carbohydrates, soluble proteins, and total lipids content were determined. Results showed that total content of carbohydrates and soluble proteins was reduced significantly, whereas the total lipids content was remarkably increased in the transgenic lines (Fig. 3c, d; Table 2). It was reported in *Chlorella vulgaris* that reduction in cellular protein concentration was correlated with the degradation of intracellular proteins to provide nitrogen source required for basic metabolic functions [29]. The previous studies have showed the role of redirecting the carbon flux from carbohydrate metabolism towards lipogenic pathway in lipid overproduction [30, 31]. Results here suggested that AGPAT1 could also play a role in redirecting the carbon flux towards the lipogenic pathway.

**Table 2:** Proportion of major cellular components in *P. tricornutum* cultured for 7 days

Cellular component	Proportion (% dry weight)		
	WT	AGPAT1-1	AGPAT1-2
Carbohydrates	14.66	9.68	8.00
Soluble proteins	30.19	20.34	20.85
Total lipids	23.43	42.98	43.49
Total	68.28	73.00	72.34

Mean values (*n* = 3) are expressed as percentage of the major cellular components

### AGPAT1 overexpression orchestrated the expression of genes in TAG pathway

The impact of overexpressed AGPAT1 on expression of other key genes such as GPAT and DGAT2 in the TAG biosynthetic pathway was evaluated. Interestingly, transcript abundance of genes such as GPAT and DGAT2 were significantly increased in AGPAT overexpressing lines compared to WT (Fig. 2e, f). It has been reported that overexpression of phosphatidylinositol synthase (PIS) resulted in abundance of substrates and thus induced the elevated transcript abundance of some key genes encoding the target enzymes involved in phosphatidylinositol signaling pathway in maize [32]. Congruently, AGPAT1 overexpression might increase AGPAT1 activity that led to higher consumption of substrates (lysophosphatidic acid) and subsequent formation of its products (PA and DAG). Thus, such unusual substrate consumption and product formation might induce the series of target enzymes related to TAG synthesis. These findings imply the principal regulatory role of AGPAT1 in metabolic alterations which is necessary for the balanced enzymatic reactions in the pathway.

### AGPAT1 overexpression elevated lipid accumulation

**Fig. 4:**
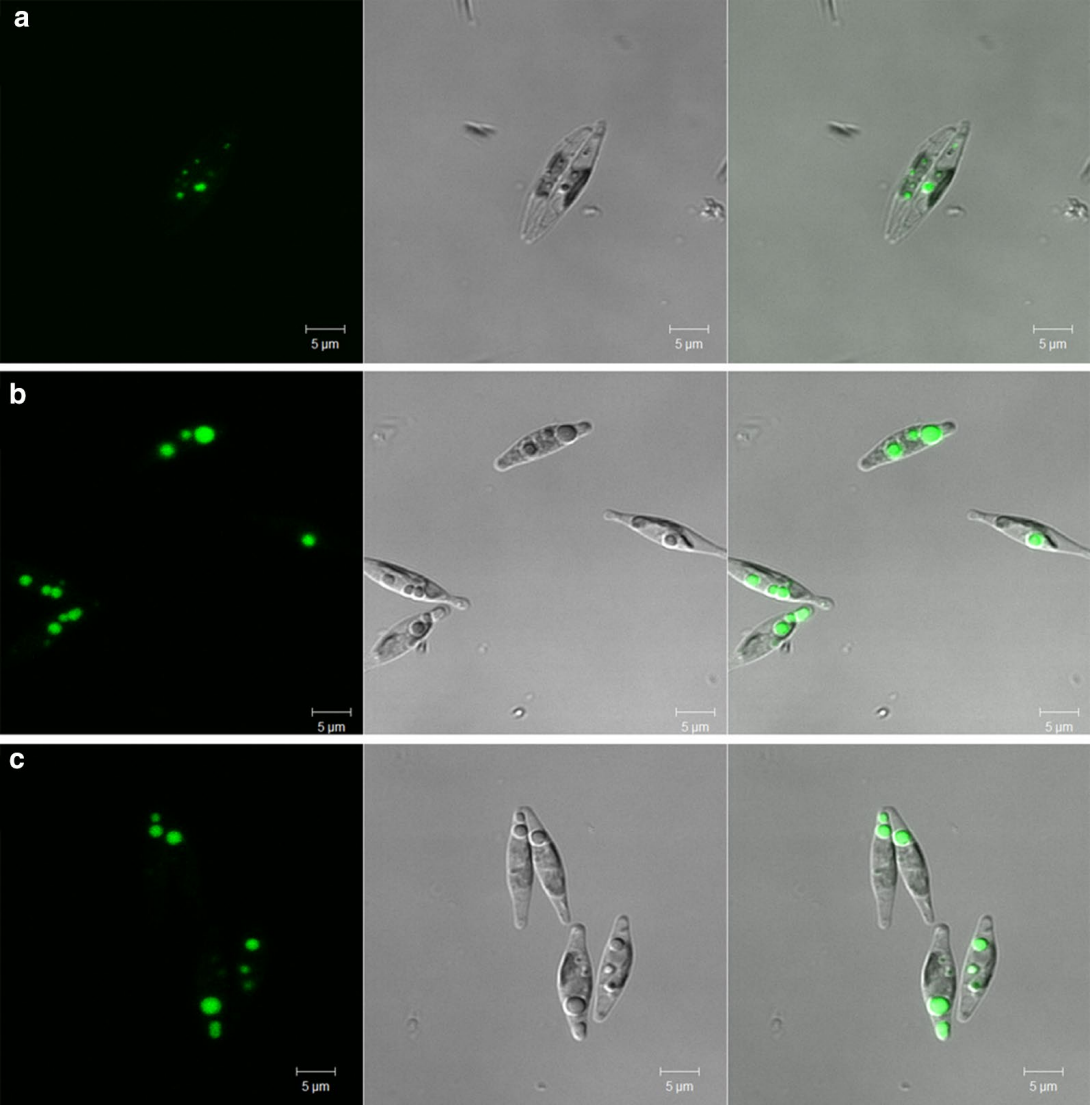
Confocal observation of Nile red stained *P. tricornutum*. **a** WT, **b**, **c** AGPAT1 transgenic lines 1 and 2. *Left panel*, fluorescence of lipid droplets; *middle panel*, differential interference contrast (DIC); *right panel*, overlay image of the *left* and *middle images*. *Bars* = 5 μm

The role of AGPAT1 overexpression was firstly evaluated on influencing the cell morphology and lipid accumulation in microalgae by Nile red staining and laser-scanning confocal microscopy. As shown in Fig. 4, the microalgae cells appeared similar size and shape, however, the volume and number of lipid droplets, and also the Nile red fluorescence were apparently higher in the transgenic lines compared to WT.

**Fig. 5:**
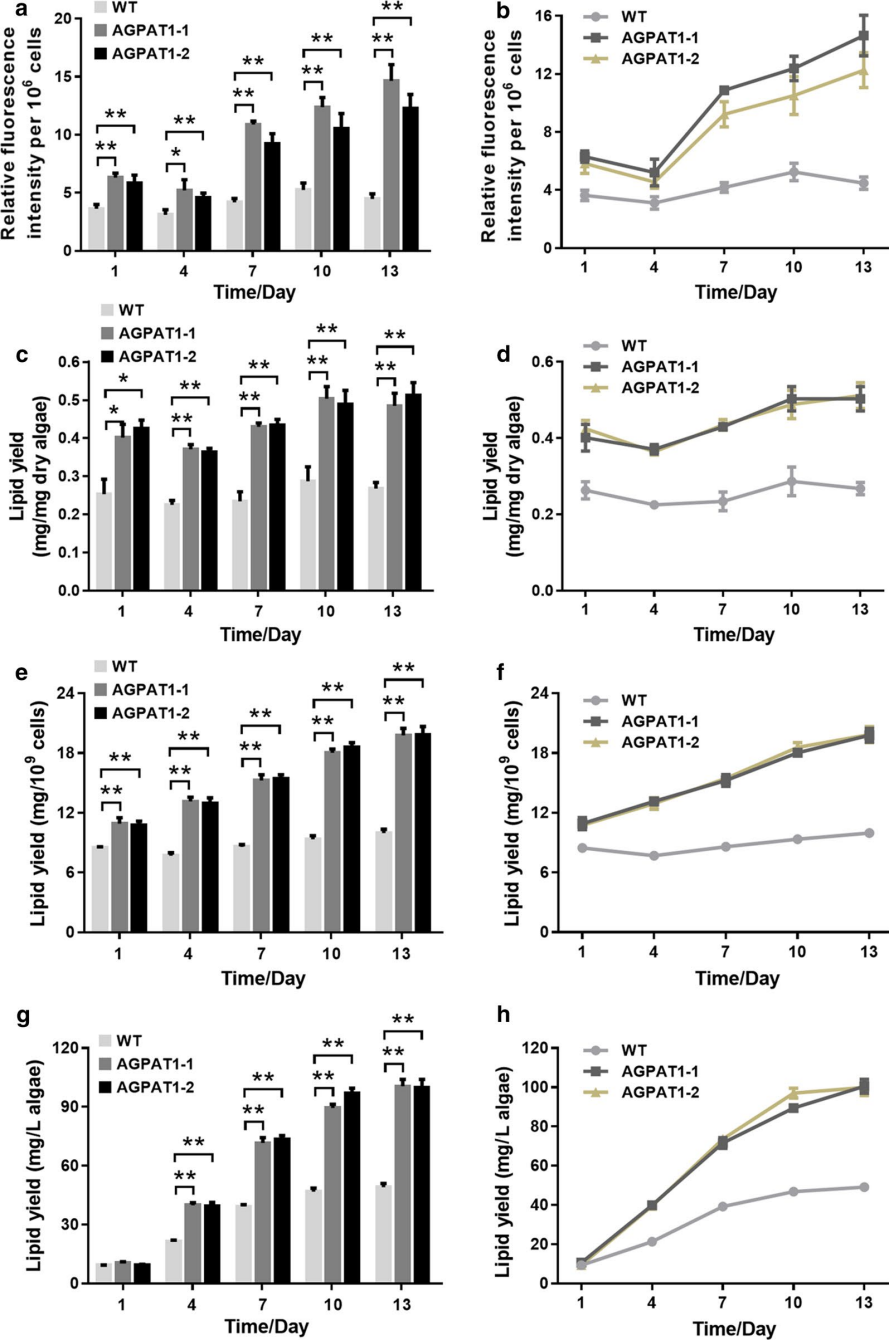
Neutral lipid content and lipid productivity in *P. tricornutum* during the whole growth cycle. **a**, **b** Relative neutral lipid content analyzed by Nile red staining showed with histogram and line diagram, respectively. **c**, **d** Lipid content per 1 mg algae (*histogram* and *line diagram*, respectively). **e**, **f** Lipid content per 10^9^ cells (*histogram* and *line diagram*, respectively). **g**, **h** Lipid content per liter culture (*histogram* and *line diagram*, respectively). Significant difference between WT and transgenic microalgae is indicated at *p < 0.05 or **p < 0.01 level. Each value represents mean ± SD (*n* = 3)

The lipid content was further quantitatively determined by both fluorometric and gravimetric analyses. Relative neutral lipid content was firstly measured by Nile red staining of microalgae during the growth period at 3-day interval. As shown in Fig. 5a and b, the relative neutral lipid content was gradually increased from the 1st day to the maximum on the 13th day in the transgenic lines. As further determined by gravimetric analysis, the lipid accumulation showed a similar trend (Fig. 5c–h). The lipid content increased gradually and reached the maximum content in the transgenic lines on the 13th day, which exhibited up to 1.81-fold higher than WT (Fig. 5c, d). The lipid yield per 10^9^ cells also reached the maximum lipid content on the 13th day and showed a 1.98-fold higher than WT (Fig. 5e, f). Likewise, the lipid yield per culture volume showed a 2.04-fold increase on the 13th day compared to WT (Fig. 5g, h). The quantitative data were consistent with the increased total volume and Nile red fluorescence of lipid droplets (Fig. 4) in transgenic lines. Metabolic engineering of microalgae for elevated neutral lipid and biomass has been considered as the prerequisite for commercial scale and economically viable biofuel production [15]. Here, AGPAT1 overexpression significantly elevated lipid accumulation without compromising algal biomass.

### AGPAT1 overexpression altered microalgal fatty acid composition

**Fig. 6:**
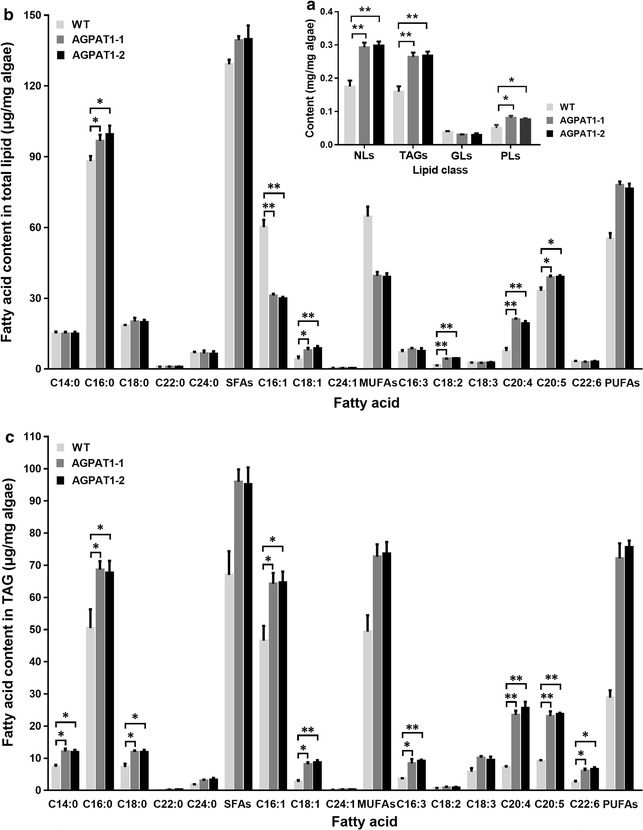
Lipid fractionation and fatty acid content in *P. tricornutum*. **a** *NL* neutral lipid, *TAG* triacylglycerol, *GL* glycolipid and *PL* phospholipid content. **b** Fatty acid profile of total lipids. **c** Fatty acid profile of triacylglycerols. Significant difference between WT and transgenic microalgae is indicated at *p < 0.05 or **p < 0.01 level. Each value represents mean ± SD (*n* = 3)

The quality and cost of biofuel could be determined mainly by the fatty acid composition accumulated in TAGs, and the functional properties of TAGs were also determined by the divergent composition of fatty acids. Therefore, we attempted to elucidate the role of AGPAT in elevating neutral lipid content and engineering the fatty acid composition in TAG of transgenic lines. Total lipids were fractionated into neutral lipids, glycolipids, and phospholipids, and the content of neutral lipid, the principal component for the generation of biofuel, was then determined gravimetrically. Neutral lipid was found to be the main component of the total lipids in both transgenic and WT lines accounting for 73.87 and 66.39% of the total lipids, respectively (Table 3). As shown in Fig. 6a, the neutral lipid content was found to be increased remarkably in transgenic lines by 67.2% than that of WT.

**Table 3:** Percentage of lipid classes in total lipids extracted from *P. tricornutum* cultured for 7 days

Lipid class	Percent (%)		
	WT	AGPAT1-1	AGPAT1-2
Neutral lipids	66.39	72.52	73.87
Glycolipids	14.42	7.53	7.32
Phospholipids	19.19	19.95	18.80

Mean values (*n* = 3) are expressed as percentage of each lipid component

In addition, fatty acid composition of lipids has been considered as a critical factor that determines the fuel properties and also the production of polyunsaturated fatty acids (PUFAs) is of significant interest in food and health care sector [33, 34]. Here, AGPAT1 overexpression altered fatty acid composition in total lipids, which enhanced the proportions of total PUFA and saturated fatty acids (SFA) and reduced monounsaturated fatty acid (MUFA) proportion (Fig. 6b; Additional file 2: Table S3). Intriguingly, the amount of C16:0 was observed to be increased significantly in the transgenic lines. In *Arabidopsis*, plastid lipids with C16:0 was found to be necessary for hormonal function [6]. These results are consistent with the catalytic role of plastidial AGPAT1 in incorporating C16:0 at the sn-2 position of lysophosphatidic acid [4, 35] and in accordance with substrate specificity of plastidial AGPAT1 towards C16:0 over C18:1.

Furthermore, to obtain the fatty acid profiling of TAGs, TAGs were separated from total lipid classes using thin-layer chromatography (TLC) and the fatty acid profile was determined. The content in algae weight was shown in Fig. 6c and the relative proportion was shown in Additional file 2: Table S4. A similar trend was found in the fatty acid data of both total lipids and TAGs, since neutral lipids were the major component of total lipids. The content in algae weight of SFAs, MUFAs, and PUFAs was all markedly elevated in transgenic lines (Fig. 6c). In terms of proportions (Additional file 2: Table S4), palmitic acid (C16:0) and palmitoleic acid (C16:1) were determined as the major components among the fatty acids, which accounted for 26.69–28.52% of TAGs in transgenic lines (Additional file 2: Table S4). The sum of SFA increased by 1.43-fold with a particular increment in C16:0. On the other hand, the sum of total MUFA was decreased in the transgenic lines. Importantly, we found that several important MUFAs and PUFAs such as C18:1, C18:2, C20:4, and C20:5 were significantly increased in the transgenic lines. Intriguingly, we found that the amount of EPA (C20:5) and DHA (C22:6) in TAGs was increased by 1.55-fold and 1.50-fold, respectively than that of WT (Fig. 6c). Increased proportion of SFAs resulted in increased ignition quality and oxidative stability of the biofuels [36], while MUFAs particularly C18:1 could improve biofuel property by increasing low-temperature property and decreasing carbon emission [37]. The elevated production of PUFAs such as C20:4 and C20:5 in the study could be due to the elevated supply of fatty acid precursors by overexpressed PtAGPAT. Bhunia et al. [38] reported that the content of fatty acids such as C20:4 and C20:5 were significantly influenced by the availability of precursor molecules. Fatty acids synthesized in the chloroplast were transported to cytosol where CoA was attached by long-chain fatty acyl-CoA synthetase and subsequent for esterification. Taken together, our findings indicate that AGPAT1 is an ideal candidate for enhancing TAG synthesis with engineered fatty acid proportion required for the synthesis of fatty acids with economical as well as health significance and also for the production of high-quality biofuel.

### AGPAT1 was targeted to the plastid and functioned in plastidial TAG synthesis

Subcellular localization of AGPAT1 was initially predicted using several online tools and further determined experimentally by immuno-electron microscopy (Additional file 1: Figure S3, Additional file 2: Table S2). As shown in Additional file 1: Figure S3, the electron dense dots representing the immuno-gold labeling against Myc-tag antibody were detected in the plastid membrane in transgenic cells, while no such labeling was detected in WT. Thus, AGPAT1 was targeted to plastid membranes. Similarly, AGPAT isoforms were reported to be localized to chloroplasts in *Arabidopsis* [6, 39], *Cuphea* [5], *Brassica napus* [6], and *Ricinus communis* [40]. Our results of AGPAT1 are in accordance with their plastidial localization.

Moreover, ultrastructural structure revealed that TAG-rich plastoglobuli were observed in the plastids of transgenic lines AGPAT-1 (Additional file 1: Figures S3c and c1) and AGPAT-2 (Additional file 1: Figures S3d and d1), respectively. Nonetheless, WT cells did not show apparent TAG-rich plastoglobuli, and they were rich in other types of granules instead (Additional file 1: Figure S3a). Except of changes in the occurrence of numerous plastoglobuli in the plastid, no other significant anatomical changes were observed. Plastoglobuli are the plastidial compartment storing lipids that were found in some higher plants to be increased during TAG accumulation and adhered to thylakoid membranes [41–44]. The findings suggested the existence of plastidial located TAG synthesis which resulted in the TAG accumulation in the plastid. It has been well known that TAG biosynthetic pathway occurs at the endoplasmic reticulum (ER) as the key enzymes related to TAG biosynthetic pathway and the synthesized TAG have been found to locate at the ER [45, 46]. However, it has been proposed in unicellular green alga *Chlamydomonas reinhardtii* a plastidial TAG pathway and the resultant TAG accumulation in plastids as well as in the cytosol as lipid droplets [4]. Congruently, in our previous report, we characterized GPAT, one of the key enzymes involved in TAG biosynthetic pathway, and it was found to localize predominantly in the chloroplast of *P. tricornutum* [2]. Despite these findings, localization of TAG biosynthetic pathway and its enzymes remain elusive in the diatom. In this study, immuno-EM revealed localization of PtAGPAT to the chloroplast membrane and the lipid-storing plastoglobuli in the plastid. Moreover, lipid analyses in transgenic lines revealed that lipid content was elevated which might be due to the additional plastid-located TAG biosynthetic mechanism.

## Conclusions

**Fig. 7:**
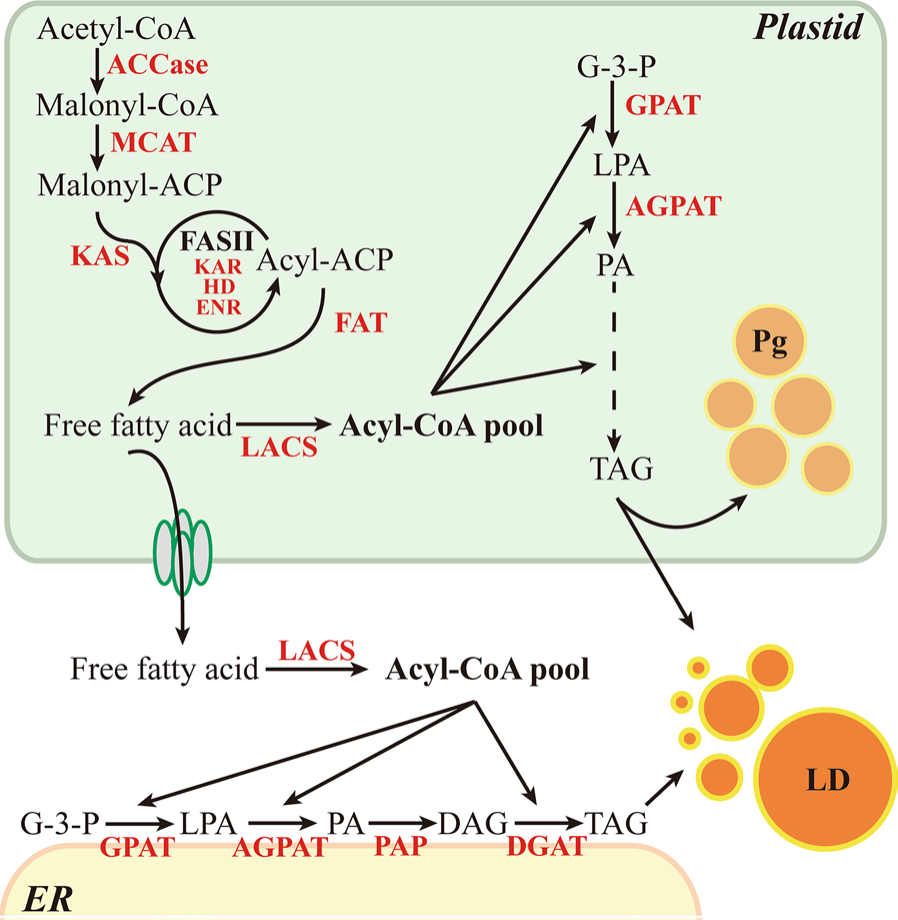
Schematic representation of plastidial TAG biosynthetic pathway in *P. tricornutum* and the regulatory role of PtAGPAT1. *Red font* represents the enzymes; *black font* represents the metabolites. *ACCase* acetyl-CoA carboxylase, *MCAT* malonyl CoA-acyl carrier protein transacylase, *KAS* 3-ketoacyl-ACP synthase; *KAR* 3-ketoacyl-ACP reductase, *HD* 3-hydroxyacyl-ACP dehydratase, *ENR* enoyl-ACP reductase, *FAT* fatty acyl-ACP thioesterase, *LACS* long-chain acyl-CoA synthetas, *GPAT* Glycerol-3-phosphate acyltransferase, *AGPAT* 1-acyl-sn-glycerol-3-phosphate acyltransferase, *PAP* phosphatidic acid phosphatase, *DGAT* diacylglycerol acyltransferase, *G*-*3*-*P* glycerol-3-phosphate, *LPA* lysophosphatidic acid, *PA* phosphatidic acid, *DAG* diacylglycerol, *TAG* triacylglycerol, *Pg* plastoglobuli, *LD* lipid droplet

In this study, a plastid-localized AGPAT1 was identified in the model diatom *P. tricornutum*. AGPAT1 overexpression elevated TAG accumulation and also altered fatty acid profile with commercial significance. The data revealed the regulatory role of plastidial AGPAT1 in elevating lipid accumulation through alternate plastid-located de novo TAG biosynthetic pathway in addition to the typical ER-located pathway in the model diatom, and also showed the role of plastidial TAG in the formation of TAG-rich plastoglobuli and cytosolic lipid droplets (Fig. 7). Overall, our data provide new insight into microalgal lipid metabolism and candidate target for metabolic engineering.

## Additional files

**Additional file 1: Figure S1.** Phylogenetic analysis and alignment of deduced amino-acid sequences of AGPAT/LPAT. **a**, Phylogenetic analysis of amino-acid sequences of the GPAT and AGPAT from several organisms. **b**, Sequence of AtLPAT1 (AT4G30580.1) was retrieved from TAIR database. RcLPAT1 (XP_002529386.1), CpuLPAT1 (ALM22868.1), and PtAGPAT1 (XP_002176893.1) were retrieved from NCBI database. Black boxes represent the acyltransferase motifs; arrowheads indicate the amino-acid residues that are not conserved. **Figure S2.** Prediction of transmembrane helix structure and topology of AGPAT1. **a**, Transmembrane helix predicted by SOSUI, TMHMM, HMMTOP, and their amino-acid sequences. **b**, Topology predicted by SOSUI. c, Topology predicted by TMHMM and HMMTOP. **Figure S3.** Subcellular localization of AGPAT1 in *P. tricornutum*. AGPAT1 was detected by immuno-gold labeling against c-Myc antibody. **a** & **b**, WT. c, AGPAT1-1. **d**, AGPAT1-2. Dense dots represent gold particles; Black arrows indicate the gold labeling of AGPAT1, the plastoglobulus (Pg) is indicated by red arrows. Ch: chloroplast; LD: lipid droplet. Bars: a, 2 μm; b, c & d, 1 μm; c1, 200 nm; d1 & d2, 100 nm.

**Additional file 2: Table S1.** Acyltransferase motifs of AGPAT1/LPAT1 from various sources. **Table S2.** In silico prediction of four AGPAT1/LPAT1. **Table S3.** Proportion of fatty acids in total lipid extracts. **Table S4.** Proportion of fatty acids in TAGs isolated by TLC.

## Abbreviations

AGPAT: 1-acyl-sn-glycerol-3-phosphate acyltransferase
GC–MS: gas chromatography–mass spectroscopy
GPAT: glycerol-3-phosphate acyltransferase
MUFA: monounsaturated fatty acid
PUFA: polyunsaturated fatty acid
qPCR: real-time quantitative PCR
SFA: saturated fatty acid
TAG: triacylglycerol
TLC: thin-layer chromatography
