# Supplementary material for: Occurrence of plastidial triacylglycerol synthesis and the potential regulatory role of AGPAT in the model diatom Phaeodactylum tricornutum
Source: Biotechnol Biofuels. 2017 Apr 20;10:97. doi: 10.1186/s13068-017-0786-0 (PMC5397801; doi:10.1186/s13068-017-0786-0)

**Additional Figures**

**Fig. S1** Phylogenetic analysis and alignment of deduced amino acid sequences of AGPAT/LPAT. **a**, Phylogenetic analysis of amino acid sequences of the GPAT and AGPAT from several organisms. **b**, Sequence of AtLPAT1 (AT4G30580.1) was retrieved from TAIR database. RcLPAT1 (XP_002529386.1), CpuLPAT1 (ALM22868.1) and PtAGPAT1 (XP_002176893.1) were retrieved from NCBI database. Black boxes represent the acyltransferase motifs; arrowheads indicate the amino acid residues that are not conserved.

**Fig. S2** Prediction of transmembrane helix structure and topology of AGPAT1. **a**, Transmembrane helix predicted by SOSUI, TMHMM, HMMTOP and their amino acid sequences. **b**, Topology predicted by SOSUI. c, Topology predicted by TMHMM and HMMTOP.

**Fig. S3** Subcellular localization of AGPAT1 in *P. tricornutum*. AGPAT1 was detected by immuno-gold labeling against c-Myc antibody. **a** & **b**, WT. c, AGPAT1-1. **d**, AGPAT1-2. Dense dots represent gold particles; Black arrows indicate the gold labeling of AGPAT1, the plastoglobulus (Pg) is indicated by red arrows. Ch: chloroplast; LD: lipid droplet. Bars: a, 2 μm; b, c & d, 1 μm; c1, 200 nm; d1 & d2, 100 nm.


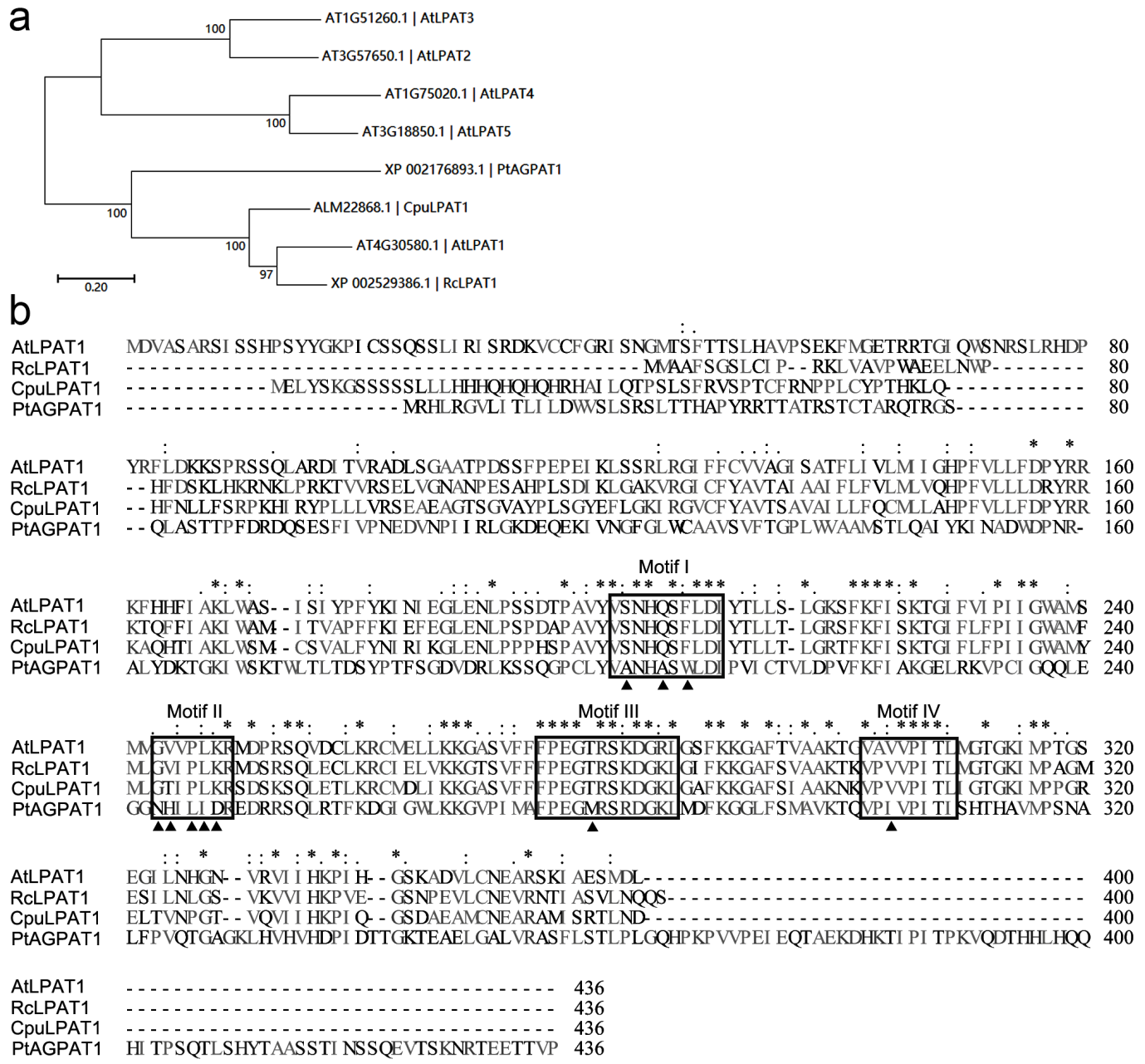


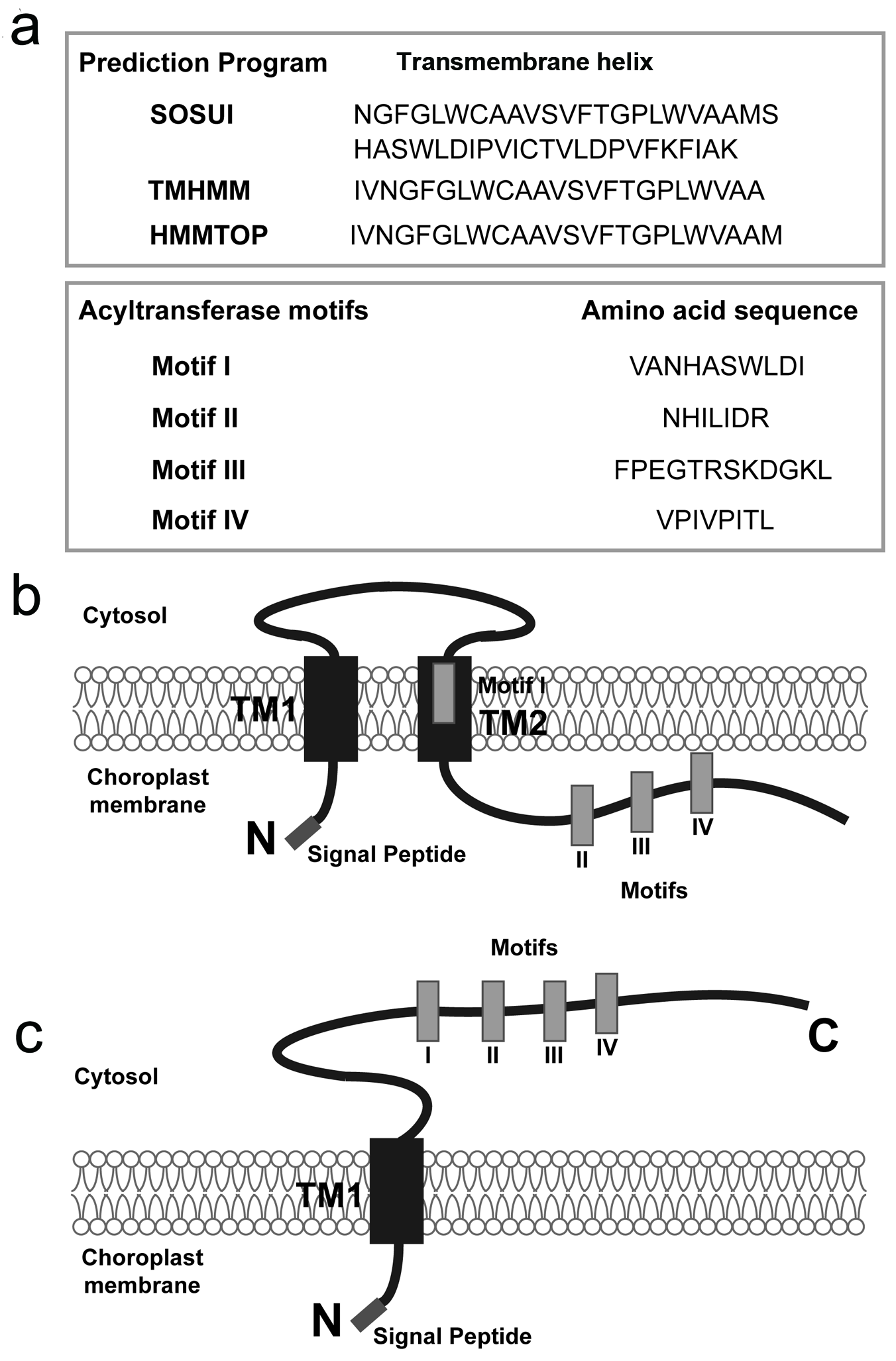


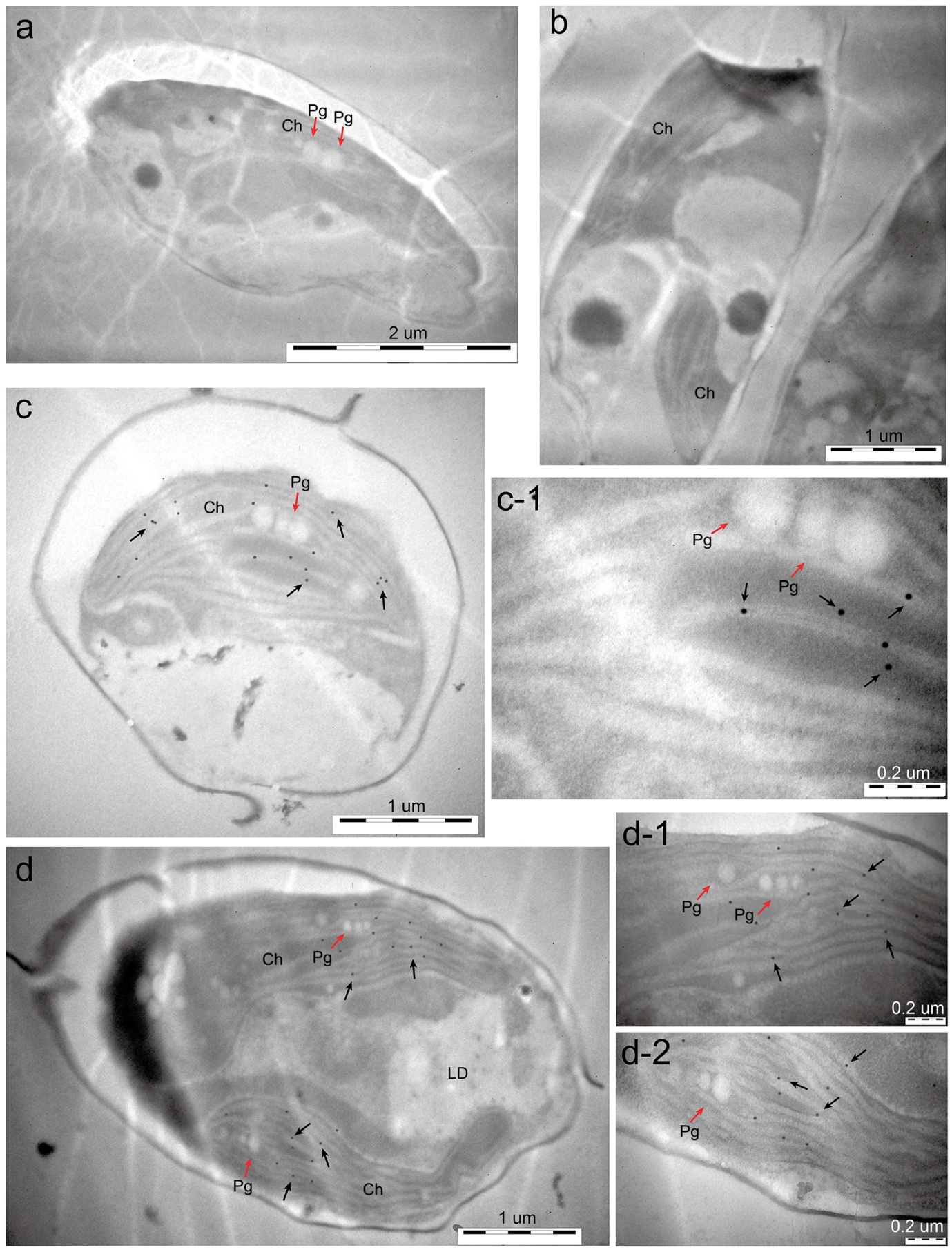

Supplement: Supplementary file 1 — Additional file 1: Figure S1. Phylogenetic analysis and alignment of deduced amino-acid sequences of AGPAT/LPAT. a, Phylogenetic analysis of amino-acid sequences of the GPAT and AGPAT from several organisms. b, Sequence of AtLPAT1 (AT4G30580.1) was retrieved from TAIR database. RcLPAT1 (XP_002529386.1), CpuLPAT1 (ALM22868.1), and PtAGPAT1 (XP_002176893.1) were retrieved from NCBI database. Black boxes represent the acyltransferase motifs; arrowheads indicate the amino-acid residues that are not conserved. Figure S2. Prediction of transmembrane helix structure and topology of AGPAT1. a, Transmembrane helix predicted by SOSUI, TMHMM, HMMTOP, and their amino-acid sequences. b, Topology predicted by SOSUI. c, Topology predicted by TMHMM and HMMTOP. Figure S3. Subcellular localization of AGPAT1 in P. tricornutum. AGPAT1 was detected by immuno-gold labeling against c-Myc antibody. a & b, WT. c, AGPAT1-1. d, AGPAT1-2. Dense dots represent gold particles; Black arrows indicate the gold labeling of AGPAT1, the plastoglobulus (Pg) is indicated by red arrows. Ch: chloroplast; LD: lipid droplet. Bars: a, 2 μm; b, c & d, 1 μm; c1, 200 nm; d1 & d2, 100 nm. [file 13068_2017_786_MOESM1_ESM.docx]
