# Supplementary material for: Occurrence of plastidial triacylglycerol synthesis and the potential regulatory role of AGPAT in the model diatom Phaeodactylum tricornutum
Source: Biotechnol Biofuels. 2017 Apr 20;10:97. doi: 10.1186/s13068-017-0786-0 (PMC5397801; doi:10.1186/s13068-017-0786-0)
Supplement: Supplementary file 2 — Additional file 2: Table S1. Acyltransferase motifs of AGPAT1/LPAT1 from various sources. Table S2. In silico prediction of four AGPAT1/LPAT1. Table S3. Proportion of fatty acids in total lipid extracts. Table S4. Proportion of fatty acids in TAGs isolated by TLC. [file 13068_2017_786_MOESM2_ESM.doc]

**Table S1** Acyltransferase motifs of AGPAT1/LPAT1 from various sources

| **Accession No.**  **(Protein ID)** | **Gene ID** | **Motif I** | **Motif II** | **Motif III** | **Motif IV** |
| --- | --- | --- | --- | --- | --- |
| XP_002176893.1 | PtAGPAT1 | VANHASWLDI | NHILIDR | FPEGMRSRDGKL | VPIVPITI |
| AT4G30580.1 | AtLPAT1 | VSNHQSFLDI | GVVPLKR | FPEGTRSKDGRL | VAVVPITL |
| ALM22868.1 | CpuLPAT1 | VSNHQSFLDI | GTIPLKR | FPEGTRSKDGKL | VPVVPITL |
| XP_002529386.1 | RcLPAT1 | VSNHQSFLDI | GVIPLKR | FPEGTRSKDGKL | VPVVPITL |

**Table S2** *In silico* prediction of four AGPAT1/LPAT1

| Gene ID | Source | Signal peptide^a^ | TargetP^b^ | LocTree3^b^ | Transmem-brane helix^c^ | Molecular weight^d^ | GRAVY^d^ |
| --- | --- | --- | --- | --- | --- | --- | --- |
| AGPAT1 | *P. tricornutum* | Yes | Chloroplast | Chloroplast membrane | 2 | 44.2kDa | -0.286 |
| AtLPAT1 | *A. thaliana* | No | Chloroplast | Chloroplast membrane | 3 | 39.4kDa | 0.030 |
| CpuLPAT1 | *C. pulcherrima* | No | Chloroplast | Chloroplast membrane | 3 | 36.9kDa | 0.038 |
| RcLPAT1 | *R. communis* | No | Cytosol | Chloroplast membrane | 4 | 33.7kDa | 0.268 |

^a^N-terminal signal peptide was predicted by SignalP; ^b^Putative subcellular localization was predicted by WoLF PSORT and LocTree3 respectively; ^c^Transmembrane helix was predicted by SOSUI; ^d^Protein molecular weight and GRARY were predicted by ProtParam.

**Table S3** Proportion of fatty acids in total lipid extracts

| **Fatty acid** | **WT** | **AGPAT1-1** | **AGPAT1-2** |
| --- | --- | --- | --- |
| C14:0 | 6.05 | 5.84 | 5.85 |
| C16:0 | 35.40 | 37.63 | 38.67 |
| C18:0 | 7.37 | 7.85 | 7.72 |
| C22:0 | 0.38 | 0.36 | 0.37 |
| C24:0 | 2.69 | 2.57 | 2.52 |
| SFAs | 51.89 | 54.26 | 55.14 |
| C16:1 | 24.14 | 12.12 | 11.62 |
| C18:1 | 1.65 | 3.10 | 3.40 |
| C24:1 | 0.16 | 0.14 | 0.14 |
| MUFAs | 25.95 | 15.37 | 15.17 |
| C16:3 | 2.85 | 3.23 | 2.97 |
| C18:2 | 0.61 | 1.71 | 1.76 |
| C18:3 | 1.08 | 1.00 | 1.05 |
| C20:4 | 3.04 | 8.20 | 7.52 |
| C20:5 | 13.32 | 15.09 | 15.20 |
| C22:6 | 1.27 | 1.13 | 1.20 |
| PUFAs | 22.16 | 30.37 | 29.70 |

Mean values (*n*=3) are expressed as percentage of each fatty acid composition.

**Table S4** Proportion of fatty acids in TAGs isolated by TLC

| **Fatty acid** | **WT** | **AGPAT1-1** | **AGPAT1-2** |
| --- | --- | --- | --- |
| C14:0 | 5.03 | 5.02 | 4.87 |
| C16:0 | 34.73 | 28.52 | 27.70 |
| C18:0 | 4.97 | 4.96 | 4.89 |
| C22:0 | 0.09 | 0.09 | 0.10 |
| C24:0 | 1.28 | 1.27 | 1.37 |
| SFAs | 46.10 | 39.87 | 38.93 |
| C16:1 | 32.09 | 26.69 | 26.46 |
| C18:1 | 1.80 | 3.37 | 3.56 |
| C24:1 | 0.12 | 0.12 | 0.12 |
| MUFAs | 34.00 | 30.18 | 30.13 |
| C16:3 | 2.59 | 3.48 | 3.76 |
| C18:2 | 0.48 | 0.38 | 0.37 |
| C18:3 | 3.98 | 4.23 | 3.89 |
| C20:4 | 4.91 | 9.75 | 10.48 |
| C20:5 | 6.30 | 9.61 | 9.71 |
| C22:6 | 1.65 | 2.51 | 2.73 |
| PUFAs | 19.90 | 29.95 | 30.94 |

Mean values (*n*=3) are expressed as percentage of each fatty acid composition.
